# Comparable Cerebral Oxygenation Patterns in Younger and Older Adults during Dual-Task Walking with Increasing Load

**DOI:** 10.3389/fnagi.2016.00240

**Published:** 2016-10-20

**Authors:** Sarah A. Fraser, Olivier Dupuy, Philippe Pouliot, Frédéric Lesage, Louis Bherer

**Affiliations:** ^1^Interdisciplinary School of Health Sciences, University of OttawaOttawa, ON, Canada; ^2^Laboratory MOVE (EA6314), Faculty of Sport Sciences, University of PoitiersPoitiers, France; ^3^Département de Génie Électrique, École Polytechnique de Montréal, MontréalQC, Canada; ^4^PERFORM Centre, Concordia UniversityMontréal, QC, Canada; ^5^Department of Medicine, Institutde Cardiologie de Montréal and University of Montréal, MontrealQC, Canada

**Keywords:** dual-task walk, fNIRS, cognitive aging, cognitive load

## Abstract

The neuroimaging literature on dual-task gait clearly demonstrates increased prefrontal cortex (PFC) involvement when performing a cognitive task while walking. However, findings from direct comparisons of the cerebral oxygenation patterns of younger (YA) and older (OA) adults during dual-task walking are mixed and it is unclear how YA and OA respond to increasing cognitive load (difficulty) while walking. This functional near infra-red (fNIRS) study examined cerebral oxygenation of YA and OA during self-paced dual-task treadmill walking at two different levels of cognitive load (auditory n-back). Changes in accuracy (%) as well as oxygenated (HbO) and deoxygenated (HbR) hemoglobin were examined. For the HbO and HbR measures, eight regions of interest (ROIs) were assessed: the anterior and posterior dorsolateral and ventrolateral PFC (aDLPFC, pDLPFC, aVLPFC, pVLPFC) in each hemisphere. Nineteen YA (*M* = 21.83 years) and 14 OA (*M* = 66.85 years) walked at a self-selected pace while performing auditory 1-back and 2-back tasks. Walking alone (single motor: SM) and performing the cognitive tasks alone (single cognitive: SC) were compared to dual-task walking (DT = SM + SC). In the behavioural data, participants were more accurate in the lowest level of load (1-back) compared to the highest (2-back; *p* < 0.001). YA were more accurate than OA overall (*p* = 0.009), and particularly in the 2-back task (*p* = 0.048). In the fNIRS data, both younger and older adults had task effects (SM < DT) in specific ROIs for ΔHbO (three YA, one OA) and ΔHbR (seven YA, eight OA). After controlling for walk speed differences, direct comparisons between YA and OA did not reveal significant age differences, but did reveal a difficulty effect in HbO in the left aDLPFC (*p* = 0.028) and significant task effects (SM < DT) in HbR for six of the eight ROIs. Findings suggest that YA and OA respond similarly to manipulations of cognitive load when walking on a treadmill at a self-selected pace.

## Introduction

Older adults (OAs) at risk of falls are more likely to stop walking when talking at the same time (Lundin-Olsson et al., 1997). This classic work and other dual-task research that combines cognitive and motor tasks (Woollacott and Shumway-Cook, 2002; Yogev-Seligmann et al., 2008; Holtzer et al., 2011; Fraser and Bherer, 2013) have demonstrated that YAs and OAs may differ in their ability to manage dual-task situations. This could partly explain greater risk for falls in older individuals. A fall in the life of an OA can lead to a cascade of negative health impacts (Masud and Morris, 2001) and large health costs (Davis et al., 2010; Heinrich et al., 2010). Understanding the behavioural and neural contributions to dual-task walk situations in order to prevent falls and provide targeted interventions is paramount.

Several studies have examined factors that can influence age-related differences in dual-task walking situations. Factors such as: cognitive or more specifically executive function contributions to gait, the input/output modality of the component tasks (i.e., visual, manual, etc.), the level of difficulty or load of component tasks (i.e., manipulations of walk speed), and task priority instructions (i.e., give equal priority to both tasks vs. sequential execution of tasks) have all influenced age-related differences in dual-task walk situations (Ble et al., 2005; Hausdorff et al., 2008; Yogev-Seligmann et al., 2008, 2010; Beurskens and Bock, 2012; Holtzer et al., 2014b; Montero-Odasso and Hachinski, 2014; Patel et al., 2014; Nascimbeni et al., 2015). While some studies suggest cognitive or motor facilitation with certain dual-task walk pairings (Fraser et al., 2007; Lövdén et al., 2008; Yogev-Seligmann et al., 2010; Li et al., 2012), in general, outcomes of dual-task walk studies have demonstrated that both younger and OAs have poorer performances when asked to walk and perform a cognitive task simultaneously in comparison to walking alone (for review see: Woollacott and Shumway-Cook, 2002; Yogev-Seligmann et al., 2008; Beurskens and Bock, 2012; Fraser and Bherer, 2013).

Neuroimaging studies examining dual-task situations and aging, using various brain imaging techniques (Holtzer et al., 2014a), have demonstrated that in aging, several brain areas are related to mobility (cerebellum, basal ganglia, parietal, and frontal cortices) and that reductions in gray and white matter volumes are associated with poor mobility outcomes. The review by Holtzer et al. (2014a) also revealed an increased involvement of the prefrontal cortex (PFC) in imagined walking and cognitively demanding walking. Neuroimaging research of gait, using the technique of functional magnetic resonance imaging (fMRI), has been limited to imagined walking (Bakker et al., 2008) or to foot/hand movements meant to reflect complex motor coordination (Heuninckx et al., 2008) because this technique is highly susceptible to movement and the fMRI scanner is a constrained space.

An alternate neuroimaging technique for quantifying brain activity during exercise or locomotion is functional near infra-red spectroscopy (fNIRS; Ekkekakis, 2009; Fabiani et al., 2014). fNIRS measures changes in blood oxygenation [oxygenated (HbO) and deoxygenated (HbR) hemoglobin] and has the advantage of being less influenced by movement than other neuroimaging techniques (Perrey, 2008). Participants wearing a fNIRS head cap while walking allows for the acquisition of brain activity during locomotion (Miyai et al., 2001; Suzuki et al., 2004; Harada et al., 2009; Holtzer et al., 2011; Mirelman et al., 2014). Of the cited fNIRS-walk studies, Harada et al. (2009) specifically examined the neural activity of OAs (59–67 years) during treadmill walking (without cognitive load). This study yielded several important research findings. Firstly, it confirmed that it is feasible to use fNIRS to explore the neural activity of gait in an older population. Secondly, the study demonstrated that an individual difference factor (i.e., high/low gait capacity) can influence PFC oxygenation (HbO) at higher walking speeds. Specifically, individuals who walk at slower speeds (low gait capacity) demonstrate greater activation in the left PFC than individuals with who walk a higher speeds (high gait capacity; walk speed > 6 km/hr). This suggests that individuals who have a slower walking pace need more PFC activity than those who have a faster walking pace to manage increased walking demands.

Functional near infra-red spectroscopy has also been applied to cognitive designs with success (Strangman et al., 2002; Arenth et al., 2007; León-Carrion et al., 2008; Herff et al., 2013; Laguë-Beauvais et al., 2013, 2015). In particular, Herff et al. (2013) have recently demonstrated that fNIRS is an appropriate technique to distinguish between different levels of mental workload (low-medium-high) in a sample of younger adults (YAs) who performed an n-back working memory task, at three different levels (1–2–3). In a more recent study, Laguë-Beauvais et al. (2015) showed that fNIRS can be used to show brain activation patterns associated with task priority instruction in a dual-task paradigm. As a neuroimaging technique, fNIRS has demonstrated the ability to accurately measure changes in cerebral oxygenation in both cognitive and motor dual-task situations.

Tanaka et al. (2014) have argued that NIRS has the specific advantage of providing simultaneous changes in cerebral concentrations of HbO and HbR during motor and cognitive tasks. In addition, the inverse relationship between decreases in HbR and increases in the blood oxygenation level dependent (BOLD) signal of fMRI is established (Ekkekakis, 2009) and offers the unique opportunity to provide converging evidence from complementary imaging techniques in cognitive-motor dual-tasks. Unfortunately, to date, most gait studies and dual-task gait studies limit their reporting to changes in concentrations of HbO (Miyai et al., 2001; Suzuki et al., 2004; Harada et al., 2009; Holtzer et al., 2011; Mirelman et al., 2014). This is in contrast to the exercise physiology literature in which reporting of HbO and HbR is common place (e.g., Ekkekakis, 2009; Rooks et al., 2010; Tempest et al., 2014; Dupuy et al., 2015; Mekari et al., 2015). In their fNIRs examination of walking while talking (WWT) at a comfortable over-ground walk pace Holtzer et al. (2011) found that both YAs and OAs demonstrated greater increases in HbO levels in the PFC when WWT in comparison to walking alone (NW). Additionally, they found a group by task interaction in which the YAs demonstrated greater increases in HbO than OAs in the WWT condition. Ohsugi et al. (2013) also found that both YA and OA show increased HbO in the PFC when a cognitive task is added to a stepping task. In addition, they found age differences in HbO such that OA demonstrated greater changes in HbO than YA but only in the post dual-task period of 10 s. Although these results are mixed, a recent study on a large cohort of healthy OAs has supported previous dual-task walk findings, such that when comparing WWT to NW bilateral increases in HbO in the PFC were found, with lateral PFC channels being more sensitive to this change (Holtzer et al., 2015). These bilateral increases in PFC have also been replicated in a sample of OAs with mild cognitive impairment (Doi et al., 2013). Research with YAs has further extended these fNIRS findings by manipulating cognitive load during dual-task gait and demonstrating that increasing the cognitive load during gait increases the HbO levels in the rostral PFC (Mirelman et al., 2014). To our knowledge no study has examined the cerebral oxygenation patterns of YAs and OAs when there are cognitive load manipulations during walking.

An overview of the fNIRS dual-task gait literature demonstrates a clear involvement of the PFC in dual-task gait in both YAs and OAs and mixed findings when the two groups are directly compared. Further, in YAs, increasing cognitive load during dual-task walking increases HbO in the PFC. Despite these important contributions to our understanding of dual-task gait in aging, several unknowns still remain. Indeed, previous studies have only presented limited data on cerebral oxygenation (HbO) and the HbR contributions to dual-task gait are unknown. The reporting of both HbO and HbR can only promote a better understanding of neural contributions during walking. Also when cognitive load is increased in an older sample, does this influence changes in cerebral oxygenation (HbO and HbR) during dual-task gait? Finally, do the changes in cerebral oxygenation differ between YAs and OAs? The goal of the current study was to extend existing fNIRS dual-walk findings by examining changes in cerebral oxygenation (both HbO and HbR) during dual-task treadmill walking under different levels of cognitive load in YAs and OAs. Based on the literature, we hypothesized: (1) that both YA and OA would demonstrate increased HbO during dual in comparison to single-task walking; (2) that both age groups would also demonstrate increased HbR (reflected by larger negative values) when comparing dual to single-task; (3) that increasing cognitive load would increase the HbO and HbR contributions in both YA and OA, but that the increase would be greater in OAs.

## Materials and Methods

### Participants

Twenty younger and 20 older right handed adults were recruited for this study. During the phone screening participants were to be excluded if unable to walk without assistance, if they had a diagnosed hearing loss and/or wore hearing aids, if they felt that chronic pain (i.e., arthritis) would affect them during 2-min walk sessions, if they had a stroke, a diagnosed cognitive impairment or any medical contraindications for low intensity exercise. None of our participants were excluded based on this phone screening. One OA who passed the screening and booked an appointment did not show-up for testing. One YA and one OA did not complete the study due to physical discomfort during testing and four OAs were unable to perform both levels of the cognitive task. Therefore, the final sample of individuals that completed the full testing procedure was 19 YA (*M*age = 21.83, *SD* = 1.92; 7 men/12 women) and 14 OA (*M*age = 66.85, *SD* = 5.26; 2 men/12 women). All participants provided written informed consent under an approved ethical board protocol from the geriatric institution where the study took place. All participants were paid an honorarium of $60 CDN for their time.

### Motor Task

All participants walked at their self-selected pace on a treadmill (Lode, Netherlands) while looking forward at a fixation cross placed at their eye level on the wall in front of the treadmill. In order to determine each participants’ self-selected pace, the speed of the treadmill was adjusted upward and downward in a stepwise fashion to determine the pace at which the participant felt was their comfortable walking pace. During this phase the participants were blinded to the speed of the treadmill while the experimenter manipulated the speed. Once a pace was determined, the participant was asked to walk for 2 min at this pace. After walking for 2 min at this pace, the participant was asked to confirm that this was indeed what they perceived as their comfortable walking pace. This pace was maintained for the entire experiment in both single-task (walk only) and dual-task (walk and n-back) conditions. For both the practice and test sessions, participants completed half of the motor trials in single-task blocks [single motor (SM)] and half with the n-back task (dual-task blocks: DTMOT). All SM and DTMOT blocks were approximately 2 min in duration. In order to control for attentional influences of gait initiation, participants began walking prior to initiating any experimental sequence or acquiring any behavioural or neuroimaging data.

### Cognitive Task: N-Back

The n-back task is a working memory task that can be parametrically manipulated to increase the memory load during testing (Jaeggi et al., 2003; Doumas et al., 2009). Typically during an n-back task, a series of stimuli are presented and the participant is asked to respond to the stimuli that they saw “n” items-back (0-back, 1-back, etc.). In the lowest load version (0-back), individuals simply have to remember and report the stimuli they just heard. As the number of items back increases, the working memory load increases placing greater attentional demands on the individual (Jaeggi et al., 2003; Doumas et al., 2009). The auditory n-back utilized in the current study was developed by our research group and has been used with OAs during dual-task gait (Fraser et al., 2014). 1-back and 2-back versions of this auditory n-back task were used. Remembering one item back was considered a low difficulty level and remembering two items back was considered a high difficulty level.

The to-be-remembered stimuli were numbers (0–9). The numbers were pseudo-randomly ordered to ensure that there were no repeats (9–9) and no ordered series (1–2–3). The numbers were recorded in a female voice and sound files (wav files) of lists of numbers were created within an E-prime 2.0 Psychology Software Tools Incorporated script. The numbers were presented through wireless headphones (Sennheiser Canada, Pointe-Claire, QC, Canada). In the 1-back version, a participant might hear the series “0 – 4 – 9 – 3…..” and once they heard 4 they would have to say 0 once they heard 9 they would have to say 4; and so on. In the 2-back version with the same list of numbers “0 – 4 – 9 – 3 …,” once the participant heard 9 they would have to say 0, once they heard 3 they would have to say 4, always keeping in mind the number heard two numbers back. The experimenter noted the participants’ responses and their accuracy (percent correct %). Single-task cognitive (SC) accuracy was equal to the participants mean performance on the n-back task while standing immobile on the treadmill. The dual-task cognitive (DTCOG) accuracy was the participants mean performance on the n-back task while walking at their self-selected pace.

A 0-back version of the task with 15 items was used in practice to ensure adequate hearing for testing. All participants reached a 90% accuracy level or greater on the number repetition baseline. In the practice phase, the single-task n-back consisted of 20 items and the dual-task n-back consisted of 29 items. During the test phase, the single-task n-back consisted of 30 items and the dual-task n-back consisted of 66 items. In practice and test phases, the stimuli were presented every 1.5 s and there was a 1 s response interval (total trial time 2.5 s).

### fNIRS Acquisition and Analysis

During the practice phase no fNIRS data was acquired. For the test runs, the 5 s baseline at the beginning of each run was used to normalize the single and dual-task data. In between each test run, the fNIRS data was saved and participants were asked to rest. This rest period between runs varied between 30–60 s and no data was acquired during this time.

Changes in concentration of oxy-hemoglobin (HbO) and deoxyhemoglobin (HbR) were measured by a multichannel, continuous-wave spectrometer (CW6, TechEn Inc., Milford, MA, USA). Eight hundred and thirty and six hundred and ninety nanometers of wavelengths were emitted in order to capture changes in light intensity are related to relative changes in hemoglobin (HbO and HbR) using the modified Beer-Lambert law (Cope et al., 1988; Villringer and Chance, 1997). Two patches, one per hemisphere, were arranged with one central, anterior–posterior row of four optodes per hemisphere. Sixteen detectors were placed strategically 2.8 cm away from the sources, eight of them were dorsal to the sources, while the other eight were ventral, so that each source had four dorsal detectors and four ventral detectors. The two patches were placed symmetrically over the lateral PFC and the most anterior and most ventral pair of source–detector of each probe (pair 8) was placed on Fp1/Fp2 using the 10/20 system (Okamoto et al., 2004). This configuration has been used repeatedly by our research group (Laguë-Beauvais et al., 2013, 2015). Following that, the 14 source–detector pairs were combined into four different approximate regions of interests (ROIs) that are based on a principal component analysis (PCA) conducted on this configuration (Laguë-Beauvais et al., 2013). They consisted of pairs 1–4 for the anterior dorsolateral PFC [DLPFC (Broadmann areas (BAs) 9, 10, and 46)], 5–7 for the posterior DLPFC (BAs 6 and 4), 8–10 for the anterior ventrolateral PFC [VLPFC (BAs 10, 45, and 46)], and 11–14 for the posterior VLPFC (BAs 4, 6, and 44) for both hemispheres.

### Procedure

The testing took place in a quiet, dark room with only ambient lighting from a shaded window which allowed participants to see the treadmill and fixation cross while limiting any saturation of the fNIRS signal from bright overhead lighting. Nasion and inion measurements were taken to correctly place the fNIRS frontal patches on each individual using the universal 10–20 system. Once the patches were placed, the signal was checked for each pair (left and right) in order to ensure no obstruction in the signal (e.g., due to hair). Then the participants underwent a task familiarization session. All conditions began with a 5-s resting baseline followed by three beeps to introduce the beginning of the task. Participants were told that the task began after the third beep which had a higher pitch. In walk trials, participants began walking prior to the initiation of the experimental task on the computer. The experimenter explained each task verbally to the participant with the lights on and then the overhead lights were shut-off during testing. For each condition (SC, SM and DT), all participants completed a practice run. During the test phase, participants completed six test runs in an ABCCBA design (single motor – single cognitive – dual-task – dual-task – single cognitive – single motor) in each level of n-back difficulty (1-back and 2-back) for a total testing time of approximately 60 min (not including instruction and rest). In dual-task conditions, the participants were told that the two tasks had equal importance and that they should do their best on both tasks. All low difficulty level (1-back) blocks were presented first, followed by the high difficulty level (2-back) to ensure the greatest familiarization with the tasks prior to the most difficult condition.

After the test session, participants completed the Digit Symbol (Strauss et al., 2006), the Trail Making tests (Strauss et al., 2006), the Digits Forward and Backward test (Wechsler, 2008). The OAs also completed the Geriatric Depression Scale (GDS; Yesavage et al., 1983) and the Mini Mental State Exam (MMSE; Folstein et al., 1975). Age differences in mean values of self-selected walk pace, performance on the 0-back task, neuropsychological data, and education level are reported in **Table 1**. The OAs in our sample had low GDS scores indicating no depression and MMSE scores above 26 (no cognitive impairment). YAs and OAs differed in their self-selected walk pace with OA walking slower than YA on average and in line with the literature (Salthouse, 1992), the two groups also differed in the number of items identified in the Digit Symbol Test (YA > OA).

**Table 1:** Mean values for the demographic and neuropsychological data of younger (YA) and older (OA) adults.

Measure	YA	OA	*p*-value
Self-selected pace (km/hr)	2.64	1.78	0.001
0-back (% correct)	100	100	n.s.
Mini Mental State Exam (MMSE) (score)	n/a	28.93	n/a
Geriatric Depression Scale (GDS) (score)	n/a	3.21	n/a
Digits Forward (score)	10.21	11.43	0.148
Digits Backward (score)	6.26	7.93	0.078
Digit Symbol (# items)	92.90	65.21	0.001
Trails B-A (secs)	25.51	36.81	0.077
Education (years)	16	15	0.229

### Statistical Analyses

#### Behavioural Data

Accuracy for the cognitive task was based on the number of correct responses (i.e., correct identification of number n-back) in all possible responses for each condition (single/dual). Cognitive accuracy the data were checked for outliers based on 2.5 standard deviations (SDs) from the group mean. No such outliers were found. Mean values for each condition were calculated for the accuracy measure. A 2 × 2 × 2 repeated measures ANOVA was conducted for the cognitive accuracy data with age group (YA/OA) as the between-subjects factor and the two within-subjects factors: task (single/dual) and difficulty (1-/2-back). Any age differences in demographic or neuropsychological data were tested with a one-way ANOVA. Alpha was set at α = 0.05 for the planned comparisons. All *post hoc* analyses were Bonferroni corrected. Please note that when discussing results associated with age, *M*_Y A_, mean younger adult; *M*_OA_, mean older adult, *SE*_Y A_, standard error younger adult, and *SE*_OA_, standard error older adult.

#### fNIRS Data

Given that YA and OA typically walk at different walk speeds (Menz et al., 2003; Yogev-Seligmann et al., 2008), we decided to first examine the changes in cerebral oxygenation in each age group separately with 2 × 2 (Task by Difficulty) ANOVAs that were conducted on the eight ROIs (four in the left hemisphere: aDLPFC, dDLPFC, aVLPFC, dDLPFC, and the same four in the right hemisphere). In addition, *t*-tests evaluating differences in cerebral oxygenation by hemisphere were also conducted on each ROI within age group. Subsequently, we directly compared cerebral changes of YA and OA in a 2 × 2 × 2 ANCOVA with the between-subjects factor (Age group), within-subjects factors (Task and Difficulty), and walk speed as a covariate. Alphas were set at α = 0.05 for main effects and *post hoc* tests were controlled for with a Bonferroni correction.

## Results

### Cognitive Accuracy

The 2 × 2 × 2 ANOVA revealed a main effect of age [*F*(1,30) = 7.83, *p* = 0.009, η^2^ = 0.21], in which YA were more accurate (*M* = 93.99%; *SE* = 2.04) than OA (*M* = 85.05%, *SE* = 2.47). There was also a main effect of difficulty [*F*(1,30) = 32.32, *p* < 0.001, η^2^ = 0.52]. Participants performed the 1-back task with greater accuracy (*M* = 96.21%, *SE* = 1.00%) than the 2-back task (*M* = 82.84%, *SE* = 2.63%). There was a marginal effect of task [*F*(1,30) = 3.86, *p* = 0.06, η^2^ = 0.11], in which single-task accuracy (*M* = 90.57%, *SE* = 1.53%) was marginally greater than dual-task accuracy (*M* = 88.47%, *SE* = 1.83%). In addition, there was a significant age by difficulty interaction [*F*(1,30) = 4.27, *p* = 0.048, η^2^ = 0.12] in which age differences in accuracy performance were greater in the 2-back condition (*M*_Y A_ = 89.74%, *SE*_Y A_ = 3.35%; *M*_OA_ = 75.94%, *SE*_OA_ = 4.05%) than the 1-back condition (*M*_Y A_ = 98.26%, *SE*_Y A_ = 1.27%; *M*_OA_ = 94.16%, *SE*_OA_ = 1.54%). Age by difficulty interaction is reported in **Table 2**.

**Table 2:** Cognitive accuracy: age by difficulty interaction.

Age group	Difficulty	Mean (%)	Standard error (%)	95% Confidence interval	
				Lower bound	Upper bound
Younger	1-back	98.26	1.27	95.67	100.86
	2-back	89.74	3.35	82.90	96.57
Older	1-back	94.16	1.54	91.02	97.29
	2-back	75.94	4.05	67.68	84.20

### fNIRS ROI Results within Age Group

As described above, general linear model results for 2 × 2 ANOVAs in the eight ROIs (four in each hemisphere) are presented in this section for each age group separately in order to examine, within age group, changes in cerebral oxygenation based on task and difficulty.

### Younger Adults

For ΔHbO, the 2 × 2 ANOVA revealed significant task effects (SM < DTMOT) in the left aVLPFC [*F*(1,18) = 5.21, *p* = 0.035, η^2^ = 0.22] in the right aDLPFC [*F*(1,18) = 4.26, *p* = 0.054, η^2^ = 0.19]; and in the right pDLPFC [*F*(1,18) = 8.33, *p* = 0.010, η^2^ = 0.32]. There were no significant difficulty or task by difficulty effects. In HbR, there were significant task effects (SM < DTMOT) in all ROIs except the right pVLPFC (*p* = 0.24), **Table 3**.

**Table 3:** Task effects in the left and right hemisphere regions of interest (ROIs) for YAs (ΔHbR).

ROI	Left hemisphere			Right hemisphere		
	*F*	*p*	η^2^	*F*	*p*	η^2^
aDLPFC	12.07	0.003	0.40	14.96	0.001	0.45
pDLPFC	11.96	0.003	0.40	5.44	0.031	0.23
aVLPFC	5.83	0.027	0.25	8.30	0.010	0.32
pVLPFC	6.83	0.018	0.28	1.49	0.239	0.08

*F = F-value, p = p-value, and η^2^ = eta squared for 2 × 2 ANOVA.*

Task effects in both the left pDLPFC [*F*(1,18) = 6.39, *p* = 0.021, η^2^ = 0.26] and left pVLPFC [*F*(1,18) = 6.26, *p* = 0.022, η^2^ = 0.26] were further qualified by a significant task by difficulty interaction in which the ΔHbR in DTMOT was greater in 1-back versus 2-back.

### Older Adults

For ΔHbO, the 2 × 2 ANOVA revealed a significant task effect (SM < DTMOT) in the left pDLPFC [*F*(1,13) = 6.80, *p* = 0.022, η^2^ = 0.34]. There was also a difficulty main effect in the right aVLPFC [*F*(1,19) = 5.72, *p* = 0.033, η^2^ = 0.31] in which the ΔHbO was greater in 2-back (*M* = 16.18, *SE* = 13.73) versus 1-back (*M* = -2.50, *SE* = 14.12). There were no significant task by difficulty interactions. In ΔHbR, there were significant task effects (SM < DTMOT) in all ROIs (**Table 4**). In addition, the right pVLPFC [*F*(1,13) = 5.19, *p* = 0.040, η^2^ = 0.29] also demonstrated significant task by difficulty effect in which the ΔHbR in DTMOT was greater in 2-back (*M* = -40.20, *SE* = 9.59) versus 1-back (*M* = -29.38, *SE* = 7.66).

**Table 4:** Task effects in the left and right hemisphere ROIs for OAs (ΔHbR).

ROI	Left hemisphere			Right hemisphere		
	*F*	*p*	η^2^	*F*	*p*	η^2^
aDLPFC	31.33	0.001	0.71	21.88	0.001	0.63
pDLPFC	26.03	0.001	0.67	17.52	0.001	0.57
aVLPFC	16.86	0.001	0.57	18.93	0.001	0.59
pVLPFC	13.78	0.003	0.52	14.90	0.002	0.53

*F = F-value, p = p-value, and η^2^ = eta squared for 2 × 2 ANOVA.*

### Hemispheric Differences

*T*-tests comparing hemisphere differences for significant ROIs within each age group revealed that in the 2-back DTMOT condition, the YAs had greater changes in ΔHbO and ΔHbR in the right pDLPFC and pVLPFC than the left. OAs did not demonstrate any significant hemisphere differences by ROI in HbO or HbR.

### fNIRS ROI Results Comparisons across Age Groups

For ΔHbO, the ANCOVAs comparing task, difficulty, and age with walk speed as a covariate revealed a significant difficulty effect [*F*(1,30) = 5.36, *p* = 0.028, η^2^ = 0.15] for the left aDLPFC, in which the ΔHbO was greater in 1-back (*M*_ΔHbO_ = 15.87, *SE* = 5.62) than in 2-back (*M*_ΔHbO_ = 13.67, *SE* = 9.39). No additional significant main effects or interactions were found. For ΔHbR, the same ANCOVA analysis revealed significant task effects (DTMOT > SM) in the left aDLPFC [*F*(1,30) = 5.52, *p* = 0.026, η^2^ = 0.16], the left pDLPFC [*F*(1,30) = 9.56, *p* = 0.004, η^2^ = 0.24], the left pVLPFC [*F*(1,30) = 8.15, *p* = 0.008, η^2^ = 0.21], the right aDLPFC [*F*(1,30) = 7.36, *p* = 0.011, η^2^ = 0.20], the right pDLPFC [*F*(1,30) = 6.00, *p* = 0.020, η^2^ = 0.17], and right aVLPFC [*F*(1,30) = 5.22, *p* = 0.030, η^2^ = 0.15]. No other significant main effects or interactions were found.

## Discussion

The goals of the current study were to examine changes in cerebral oxygenation (both HbO and HbR) and cognitive accuracy during dual-task walking under different difficulty levels and explore any age differences in dual-task treadmill walking at a self-selected pace. Difficulty effects were found in both the behavioural and cerebral oxygenation data. When examining the cerebral oxygenation patterns of the two age groups separately, both YAs and OAs had task effects (SM < DTMOT) in specific ROI for ΔHbO (three YA and one OA) and ΔHbR (seven YA, eight OA). After controlling for differences in self-selected walk speed, direct comparisons of the cerebral oxygenation patterns of YAs and OAs revealed difficulty effect in one ROI (left aDLPFC) for ΔHbO, task effects in six ROIs for ΔHbR and did not reveal any significant age differences or interactions.

In the behavioural data, YA were more accurate overall than OA and both groups responded more accurately to the 1-back than the 2-back. There was also an interaction between group and difficulty such that age differences in accuracy were greater in the 2-back in comparison to the 1-back. These effects support our difficulty manipulation in that participants made more errors in the more difficult 2-back condition than the 1-back condition. In addition, the increased difficulty resulted in more response errors for the OAs than the YAs, suggesting that the load manipulation had a greater effect on OAs performance than YAs, a finding that is in line with the literature on age-related differences in dual-task walking (Woollacott and Shumway-Cook, 2002). Interestingly, in the accuracy data, there were no significant main effects of task or interactions with task suggesting that both the YA and the OA were able to maintain their single-task accuracy levels in the dual-task condition.

When examining the changes in cerebral oxygenation within each age group, YAs demonstrated task effects (SM < DTMOT) in three out of the eight ROIs for ΔHbO and in seven out of the eight ROIs for ΔHbR. Although there are only select ROIs implicated in this task effect, less channels than those reported in over-ground walking (Holtzer et al., 2011), the direction of the effect is the same and supports a greater involvement of PFC when dual-task walking on a treadmill at a self-selected pace. Task by difficulty interactions found in YA, in two ROIs in the left hemisphere (posterior DLPFC and VLPFC), suggest that YA had greater changes in HbR in the dual-task 1-back condition when compared to the dual-task 2-back condition. In fMRI research with YAs and n-back load manipulations, Jaeggi et al. (2007) also found left lateralized PFC contributions to load manipulations of the n-back task. The paradox of finding a larger HbR change in the 1-back condition in comparison to the 2-back condition may be suggestive of a learning effect in the YAs, such that the presentation of the 1-back condition first may have improved YAs ability to manage the dual-task when subsequently presented with the 2-back condition. This is in line with fNIRS motor skills learning research that has demonstrated the magnitude of the change in cortical oxygenation attenuates at the same time as objective behavioural improvements on the task (for review: Leff et al., 2011).

Older adults demonstrated the same task effect (SM < DTMOT) in ΔHbO in the left pDLPFC, in all eight ROIs in ΔHbR and a difficulty effect in the right aVLPFC in which there was a greater change in HbO during dual-task walking in the 2-back condition than in the 1-back condition. In line with the literature, OAs demonstrate increases in HbO during dual-task walking in comparison to walking alone (Holtzer et al., 2011, 2015). In comparison to previous fNIRS studies with OAs, fewer ROIs (or a lower number of channels) are implicated in the task effect in the current study. This may be due the type of walking demands (over-ground vs. treadmill walking) or, that in our study, the preferred walk speed was chosen at the beginning and had to be maintained throughout the blocks. Unlike over-ground dual-task walk paradigms (Holtzer et al., 2011, 2015), OAs in the present study could not adapt their gait speed in response to cognitive demands.

After controlling for differences in walk speed, no significant age differences emerged but several significant task effects (SM < DTMOT; for 6/8 ROIs) remained in ΔHbR and one main effect of difficulty (1-back > 2-back) emerged in ΔHbO, for the left aDLPFC. Interestingly, the left aDLPFC demonstrates a concomitant decrease in HbR. In the current study, the left aDLPFC emerges as an area that demonstrates a difficulty effect and the task effect for both age groups after controlling for walk speed. Based on the literature, the aDLPFC ROI defined in this paper, would contain BA46 which has been implicated in dual-task performances (Smith and Jonides, 1999; Laguë-Beauvais et al., 2015). The region of the difficulty effect found in the left aDLPFC (ΔHbO) aligns well with findings from the cognitive load manipulation of Mirelman et al. (2014). They also identified the rostral PFC as important to simultaneous processing of cognitive and motor tasks and demonstrated graded increases in HbO activity with increasing difficulty levels (Mirelman et al., 2014). One interpretation of the decrease in HbO from 1- to 2-back could be that, in the present design, younger participants benefited from the presentation order (1-back then 2-back) and that this resulted in a smaller change in HbO in the condition with the higher load. The OA findings suggest that OAs did not benefit from the order of presentation as they demonstrate a difficulty effect for HbO in the expected direction (1-back < 2-back).

In the large ANCOVA controlling for walk speed, several significant task effects are maintained in HbR and this raises the question as to the importance of this measure in the dual-task walk literature. It may be the case that in the present design, HbR captures changes that are not as evident in HbO when asking participants to walk on a treadmill at a self-selected pace. In addition, given the inverse relationship between HbR and the BOLD signal, these significant changes in HbR (larger decreases) could be representative of increased activations in fMRI but additional research combining these methods is needed to further explore the contributions of HbR to the dual-task gait literature and to rule-out physiological noise influencing the HbR variable.

Theoretically, the present results fit with resource based or capacity sharing models from the dual-task literature which suggest that when the task demands exceed the capacity of the system dual-task costs may be incurred (Fraser and Bherer, 2013). Performance on one or both tasks may decline and/or changes in brain activity between single and dual-task may be observed (Fraser and Bherer, 2013). One important finding to note is that none of our participants demonstrated behavioural dual-task costs (they maintained accuracy levels from single to dual-task) but the participants did demonstrate changes in cerebral oxygenation in response to dual-task demands. Greater changes in HbO and HbR in dual-task conditions in the PFC without behavioural costs also fits with the compensation-related utilization of neural circuits hypothesis (CRUNCH; Reuter-Lorenz and Lustig, 2005). The participants in our sample may have maintained their accuracy from single to dual-task walking by increasing their cerebral activity in response to cognitive load. This interpretation would be in line with recent electroencephalogram findings (De Sanctis et al., 2014) that also found changes in neural activity with increased task load and a lack of dual-task performance costs. In our study, the YAs appear to be better at utilizing their cerebral activity to maintain task performance, as they demonstrate a decrease in cerebral activity at higher loads possibly due to dual-task exposure in the lower load condition. The OAs show the opposite effect (increased activity based on load), but direct comparisons in our sample cannot confirm this possibility. Additional fNIRS dual-task walk research with cognitive load manipulations in younger and OAs is needed to test the behavioral and cerebral contributions proposed in CRUNCH.

### Limitations

In the current design, the order of presentation was purposely set so that all participants would be exposed to the easier difficulty level (1-back) first and then they would perform the more cognitively demanding 2-back task. This design allowed for both YAs and OAs to have experience with the two tasks prior to tackling the more difficult condition which minimizes any differences that would be due to not understanding the component tasks. However, it is likely that our choice to present the n-back tasks this way may have minimized the task effects in dual-task performance.

It is well known that fNIRS has greater temporal specificity and less spatial specificity than fMRI. As such, despite the many regions involved in walking and dual-tasking, the choice of the current neuroimaging technique limits our ROI to the cortical surface of the PFC. Complementary neuroimaging techniques (fMRI, EEG) and fNIRS helmets that are designed to cover larger areas (including more motor areas) will help elucidate contributions from both cognitive and motor areas during dual-task walking.

## Conclusion

The results of this research study support the findings of the existing dual-task gait in the fNIRS literature and extend earlier ΔHbO findings to include changes in deoxygenated hemoglobin (HbR) during walking. The present findings suggest that OAs with adequate task exposure can respond to cognitively demanding tasks while walking in a similar fashion to their younger counterparts. The differences in the cerebral oxygenation of each age group examined separately suggest that perhaps, in the HbR measure, the YAs benefited from the additional training demonstrated a smaller change in the dual-task with the higher difficulty level in comparison to the lower difficulty level, whereas the OAs demonstrated the opposite pattern in HbO (increased activity to the more difficult task). However, after controlling for age differences in walk speed, direct statistical testing of the two age groups demonstrated a lack of age differences in cerebral activity during dual-task walking. Continued research comparing YAs and OAs with fNIRS in ecological dual-task walking situations involving manipulations in cognitive load will help clarify which dual-task situations and what types of responses to dual-task situations could increase fall risk in OAs.

## Author Contributions

SF designed, trained, and supervised the data collection of this experiment with several undergraduates and one graduate student. SF and OD worked on the raw data and interpretations of the findings. PP and FL aided with the signal processing analysis, and filtering of the raw NIRS data. All the equipment (including NIRS Techen device) and lab space was provided by LB. All co-authors have read and provided feedback and improvements to several iterations of this manuscript in order to arrive at the current submission.

## Conflict of Interest Statement

The authors declare that the research was conducted in the absence of any commercial or financial relationships that could be construed as a potential conflict of interest.
